# The Question of the Extermination of Cattle-tuberculosis

**Published:** 1898-08

**Authors:** 


THE QUESTION OF THE EXTERMINATION OF CATTLE-TUBERCULOSIS. The continued spread of cattle-tuberculosis and its intimate causal relation to human tuberculosis makes its extermination one of the principal objects of contemporary legislation. District veterinarian Rudovsky was appointed to make a report on this subject at the Austrian Agrarian Assembly. Although we have not the full text of his report, yet we gather the gist of it from the Wr. agricultural newspaper of February 12th.

In the district of Muhren, under Rudovsky's direction, some 2314 cattle were inoculated with tuberculin; 39.84 per cent. proved to be tuberculous; 4.40 per cent. were suspected of it, and 55.75 per cent. were free from all tuberculous symptoms. Through these inoculations the well-known fact was confirmed that tuberculosis is much more widely spread in large herds than in smaller ones; that the frequency of tuberculosis increases with the age of the animal, and that acclimated species show the smallest percentage in tuberculous statistics. We heartily agree with Dr. Rudovsky when he emphasizes the necessity of immediately tackling the problem of extermination and confirms the great diagnostic value of tuberculin.

On the other hand, we cannot agree with several points in his report. He asserts that tuberculosis is not inherited from parents, and is present only "where the excrement containing bacilli of tuberculous men and animals is to be found." He overlooks the fact that the undeveloped fœtus of tuberculous cows have, after slaughter, been repeatedly found to be tuberculous. Inheritance, therefore, plays some if not a very important rôle in the spread of the disease.

We agree, further, with him in the assertion that it will be possible to exterminate tuberculosis, just as other infectious diseases; but he stands in contradiction to the experience of everyone that recognizes the innumerable ways of infection and the immense spread of the disease, when he asserts that the extermination of tuberculosis can be accomplished without great sacrifice on the part of the public treasury. This view is too sanguine, and leads the public to the erroneous opinion that the government is purposely avoiding the question of tuberculosis extermination, and is acting contrary to the most vital agricultural interests.

Veterinarians have on all occasions called attention to the great importance of tuberculin reaction, but at the same time they have always emphasized the fact that tuberculin-inoculation will never be practicable without very material aid from the State in the shape of remuneration for slaughtered animals. For this purpose a sum will be necessary in comparison with which the sum already expended in exterminating pulmonary consumption is nothing, and at the same time the agricultural population must likewise be willing to make some sacrifice, which is not the case to-day.

It is a matter of indifference to us if we are again characterized as volunteer directors of the government when we assert that tuberculosis extermination, in spite of a great financial sacrifice on the part of the State, will not be possible so long as the agricultural classes of Austria are not willing to have some general system of compulsory cattle-insurance carried out. Alas, we seem to-day further away from this point of view than ever.—*Idem.*

